# The Repetitive Oligopeptide Sequences Modulate Cytopathic Potency but Are Not Crucial for Cellular Uptake of *Clostridium difficile* Toxin A

**DOI:** 10.1371/journal.pone.0017623

**Published:** 2011-03-18

**Authors:** Alexandra Olling, Sebastian Goy, Florian Hoffmann, Helma Tatge, Ingo Just, Ralf Gerhard

**Affiliations:** Institut für Toxikologie, Medizinische Hochschule Hannover, Hannover, Germany; Indian Institute of Science, India

## Abstract

The pathogenicity of *Clostridium difficile* is primarily linked to secretion of the intracellular acting toxins A (TcdA) and B (TcdB) which monoglucosylate and thereby inactivate Rho GTPases of host cells. Although the molecular mode of action of TcdA and TcdB is well understood, far less is known about toxin binding and uptake. It is acknowledged that the C-terminally combined repetitive oligopeptides (CROPs) of the toxins function as receptor binding domain. The current study evaluates the role of the CROP domain with respect to functionality of TcdA and TcdB. Therefore, we generated truncated TcdA devoid of the CROPs (TcdA^1–1874^) and found that this mutant was still cytopathic. However, TcdA^1–1874^ possesses about 5 to 10-fold less potency towards 3T3 and HT29 cells compared to the full length toxin. Interestingly, CHO-C6 cells even showed almost identical susceptibility towards truncated and full length TcdA concerning Rac1 glucosylation or cell rounding, respectively. FACS and Western blot analyses elucidated these differences and revealed a correlation between CROP-binding to the cell surface and toxin potency. These findings refute the accepted opinion of solely CROP- mediated toxin internalization. Competition experiments demonstrated that presence neither of TcdA CROPs nor of full length TcdA reduced binding of truncated TcdA^1–1874^ to HT29 cells. We assume that toxin uptake might additionally occur through alternative receptor structures and/or other associated endocytotic pathways. The second assumption was substantiated by TER measurements showing that basolaterally applied TcdA^1–1874^ exhibits considerably higher cytotoxic potency than apically applied mutant or even full length TcdA, the latter being almost independent of the side of application. Thus, different routes for cellular uptake might enable the toxins to enter a broader repertoire of cell types leading to the observed multifarious pathogenesis of *C. difficile*.

## Introduction

*Clostridium difficile* associated disease is primarily linked to the production of the two homologous pathogenicity factors toxin A (TcdA) and toxin B (TcdB). Both toxins are members of the family of large clostridial glucosyltransferases that monoglucosylate small GTP-binding proteins of the Rho family [1]. Glucosylation of Rho GTPases renders these proteins in their inactive state leading to breakdown of the actin cytoskeleton with subsequent cell rounding. In combination with ELISA this cell rounding assay, also referred to as cytotoxicity assay, is still gold standard when performed on Vero cells for diagnosis of pathogenic *C. difficile* infection.

The toxins are single chain proteins of an A/B type structure where the catalytic active glucosyltransferase domain is located at the N-terminus and the proposed receptor binding domain at the C-terminus [2]. The C-terminus of TcdA and TcdB consists of 37 or 19 repeats, respectively, building combined repetitive oligopeptide structures (CROPs) [3]–[6] from which it is known that they bind to carbohydrate structures. Detailed studies were performed in the early 1990s and Galα1-3Galβ1-4βGlcNAc was described as binding structure for TcdA. Since this oligosaccharide is not present in humans, at least a type 2-core with a β1–4 linkage (Galβ1–4βGlcNAc) is essential, which is found on the carbohydrate antigens I, X, and Y [7]. Additionally, the C-terminal repeats bind Ca^2+^ thereby enhancing potency of TcdA [8]. Despite the respective carbohydrate structure few is known about the nature of the receptor. Sucrase- isomaltase as well as the glycoprotein gp96 have been suggested as functional binding proteins or receptor for TcdA [9], [10]. The entry of TcdA and TcdB into the target cell is mediated by binding to their receptors which triggers endocytosis. Although the functional receptors for TcdA and TcdB have not been definitely identified, both toxins seem to have different receptors. The crucial step for pathogenicity of the toxins is the translocation of the catalytic domains into the cytosol of target cells. Acidification of the endosomal vesicular lumen induces conformational changes of the toxins which allows the insertion into the vesicle membrane and translocation of the N-terminally located catalytic glucosyltransferase (GT) domain into the cytosol. The GT-domain is autoproteolytically released from the trunk by a toxin-inherent cysteine protease domain [11], [12].

In 2007, Amimoto and co-workers reported on a novel toxin homologous to large clostridial glucosylating toxins that is produced by *C. perfringens* type C strains [13]. Interestingly, this toxin lacks the repetitive combined oligopeptide sequences that are supposed to function as receptor-binding structures, but still displays cytotoxic activity. Based on this finding we dispute the necessity of the CROP domain concerning functional properties of TcdA and TcdB. The current study evaluates the functional role of the TcdA CROP-domain by utilizing truncated TcdA where the C-terminal amino acids 1875–2710 were deleted (TcdA^1–1874^). We proved that the C-terminal repeats are not essential for TcdA function albeit they determine the potency of the toxin by interacting with surface structures of host cells.

## Materials and Methods

### Chemicals and reagents

The antibodies used were: polyclonal rabbit antibodies α-TcdA_1–543_, α-TcdA_1–1065_, and α-TcdA_1–2710_ (Institute of Toxicology, Hannover Medical School); α-TcdA_1875–2710_ (this study); monoclonal anti-Rac1 antibody (clone 102; BD PharMingen) recognizing total Rac1; monoclonal anti-Rac1 antibody (clone 23A8; Upstate) recognizing non-glucosylated Rac1; antibody against β-Actin (clone AC15) was from Sigma; α-EEA1 was purchased from BD Transduction Laboratories; horseradish-conjugated goat anti-mouse IgG and goat anti-rabbit IgG were from Rockland Immunochemicals and Bafilomycin A1 from Sigma. The *Bacillus megaterium* expression system was from MoBiTec. Fluorescent Protein Labeling Kits Lightning-Link™PE/Cy5 and Lightning-Link™Atto488 were purchased from Innova Biosciences. All chemicals were of the highest purity available.

### Expression of recombinant toxins

The *C. difficile* toxins (strain VPI 10463, GenBank accession no. X51797) were recombinantly expressed in the *B. megaterium* expression system as His-tagged fusion proteins (MoBiTec, Germany). Expression and purification was performed after standard protocol as described previously (Burger et al., 2003). Truncated TcdA (amino acids 1–1874) was generated by using a specific endonuclease recognition site (bp 5620) in TcdA, which is located within the first repetitive sequence, and cloning of the resulting toxin fragment into the modified *B. megaterium* expression vector pWH1520 [14]. This SpeI recognition site was also used to mobilize and thereby eliminate bases 1–5622 from the TcdA-encoding plasmid. Re-ligation of the remaining construct resulted in generation of pWH-TcdA_5623–8130_ encoding the amino acids 1875–2710 that correspond to the TcdA CROP domain. The purified expression product was used for immunization.

The TcdA mutant containing the complete N-terminal domain and hydrophobic region (TcdA^1–1101^) was generated by extension of construct pWH-TcdA_1–3195_ encoding amino acids 1–1065 as described previously (Teichert *et. al*, 2006). Therefore, a synthesized oligonucleotide encompassing the *tcdA* base pairs encoding amino acids 1066–1101 (sense: 5′- TAAGGTGGGTGTTTTAGCAATAAATATGTCATTATCTATAGCTGCAACTGTAG CTTCAATTGTTGGAATAGGTGCTGAAGTTACTATTTTCTTATTACCTATAGCTGGTATAGGACATCATCATCATCATCATTAGA-3′; antisense: 5′- GATCTCTAATGATGAT GATGATGATGTCCTATACCAGCTATAGGTAATAAGAAAATAGTAACTTCAGCACCTATTCCAACAATTGAAGCTACAGTTGCAGCTATAGATAATGACATATTTATTGCTAAAACACCCACC-3′) were purchased from Biomers (Germany) and ligated into construct pWH-TcdA_1–3195_ by Bpu10 and BglII restriction sites.

To generate EGFP-labelled CROP domain the *egfp* gene was amplified from vector pEGFP-C1 (BD Biosciences Clontech) and inserted at the SpeI site of construct pWH-TcdA_5623–8130_.

The *tcdB* gene was amplified from *C. difficile* (VPI 10463) chromosomal DNA using forward primer 5'-AGTCTGTACAATGAGTTTAGTTAATAG-3′ and reverse primer 5′-AGTCAG ATCTCTTCACTAATCACTAATTG-3′. The PCR product was digested by BsrGI and BamHI enzymes and ligated into pHis1522 vector (MoBiTec, Goettingen, Germany) for production of full length TcdB. Generation of the construct encoding the truncated TcdB^1–1852^ was achieved by two cloning steps. Mobilization of the *tcdB* bases 1–5260 was accomplished by BsrGI and SpeI restriction digest from the full length construct pHis1522-TcdB followed by ligation of the fragment into vector pHis1522. To replenish construct pHis1522-TcdB_1–5260_ up to base pair 5556, bases 5261–5556 were amplified (forward primer: 5′-AGCTACTAGTGAAGAAAATAAGGTGTCACAAG-3′; reverse primer: 5′-AGCTGGA TCCCCAAATTATTTACTGGTGGTTTA-3′) and ligated into pHis1522-TcdB_1-5260_ through SpeI and BamHI restriction sites. The resulting construct pHis1522-TcdB_1–5556_ encodes the C-terminal truncated TcdB^1–1852^. All constructs were sequenced.

### Generation of specific antibody

Immunization of a female New Zealand rabbit was performed after standard protocol using the affinity purified immunogen TcdA^1875–2710^ (Permission No. 33-42502-03A351, see *Ethics statement*). First immunisation was performed with 100 µg of protein followed by a single boost after four weeks. Blood was collected three weeks after boost immunisation. Specificity of anti-serum was checked by Western blot using the antigen as control.

### Cell culture and cytotoxicity assay

3T3 mouse fibroblasts and the human colon carcinoma cell CaCo-2 were cultivated under standard conditions in Dulbeccos' modified Eagle's medium (DMEM) supplemented with 10% foetal bovine serum (FBS), 100 µM penicillin, 100 µg/ml streptomycin, and for CaCo-2 cells only 1% non-essential amino acids (NEA) [15], [16]. The human colonic crypt cell line HT29 was grown in DMEM/Ham's F-12 supplemented with 10% fetal bovine serum, 100 µM penicillin and 100 µg/ml streptomycin. The hamster ovar cells CHO-C6 were cultivated in DMEM/Ham's F-12 supplemented with 5% fetal bovine serum, 1 mM sodium pyruvate, 100 µM penicillin and 100 µg/ml streptomycin [17]. For cytotoxicity assay, cells were seeded in 24-well chambers and grown for 24 hours to sub-confluence. Toxins were diluted in the respective medium and indicated concentrations were added in appropriate volumes to the indicated cells. For inhibitor experiments, 3T3 fibroblasts were pre-treated with Bafilomycin A1 (100 nM) for 5 min followed by application of indicated toxins or toxin fragments, respectively. Toxin-induced cell rounding was monitored by light microscopy and cytopathic effect (CPE) was quantified as round cells per total cells. The cell lysates of toxin-treated cells were subjected to SDS-PAGE and Western blot analysis to determine status of Rac1-glucosyalation as direct marker for intracellular action of toxins. Specific antibodies were used recognizing either only non-glucosylated Rac1 (clone 23A8) or total Rac1 (clone 102). For internalization assays, Bafilomycin A1 was applied to 3T3 fibroblasts at indicated time points in relation to toxin treatment and the cytopathic effect was determined as described above.

### Fluorescent Toxin Labeling

TcdA and TcdA^1–1874^ were labeled with Lightning-Link™ PE-Cy5 and Lightning-Link™ Atto188, respectively, according to the manufacture's instructions. In brief, the lyophilized fluorophor was dissolved in toxin solution supplemented with LL-Modifier reagent. Conjugation reaction was performed for 3 h at RT and stopped by addition of LL-Quencher FD reagent. TcdA was conjugated to the tandem fluorophor PE/Cy5 and TcdA^1–1874^ to the fluorescent dye Atto488 giving a toxin concentration of 2–5 µM.

### Binding assay and Fluorescence-activated cell sorting (FACS)

For comparative binding studies to intact cells, cells grown in 24-well chambers were incubated for 30 min on ice with 6 nM of TcdA or TcdA^1–1874^. Cells were washed twice in phosphate-buffered saline (PBS) to eliminate non-bound toxins and lyzed in 1x Lämmli, supplemented with 100 µM 4-(2-Aminoethyl)-benzensulfonylfluorid (AEBSF). Lysates were subjected to SDS-PAGE and Western blot analysis to detect the level of bound toxins.

Binding of the CROPs and the toxins TcdA and TcdA^1–1874^ to the cell surface was additionally analyzed by flow cytometry. Therefore, adherent cells were suspended by Accutase treatment and 500,000 cells were incubated at 4°C for 30 min with indicated concentrations of EGFP, EGFP-fused TcdA^1875–2710^ or fluorescent labeled TcdA (TcdA-PE/Cy5) and TcdA^1–1874^ (TcdA^1–1874^-Atto488), respectively. Under standard conditions cells were washed twice with ice-cold PBS by centrifugation at 200 g for 5 min at 4°C to eliminate non-bound toxins. Except for fluorescent labeled toxins (TcdA-PE/Cy5 and TcdA^1–1874^-Atto488) cells were subsequently fixed in 4% of formaldehyde solution, repeatedly washed and finally subjected to flow cytometry (FACScan flow cytometer; Becton Dickinson). Ten thousand events were monitored per condition.

For competition assays 500,000 HT29 cells were pre-incubated for 30 min at 4°C in 25-fold excess with indicated concentrations of non-labeled TcdA, TcdA^1–1874^ or TcdA^1875–2710^, respectively, to saturate all binding sites. Except when otherwise described, cells were washed to eliminate non-bound protein and incubated with either PE/Cy5-labeled TcdA, Atto488-conjugated TcdA^1–1874^ or both or EGFP-fused TcdA^1875–2710^, respectively. Toxins were applied to the cells at indicated concentrations and fluorescence activity was monitored with and without pre-treatment as described above.

### Immunofluorescence microscopy

HT29 cells grown on coverslips were incubated on ice for 30 min with 80 nM of EGFP or EGFP-labelled TcdA^1875–2710^, respectively, followed by several washing-steps with ice cold PBS. Cellular uptake was allowed by addition of 37°C-warmed PBS supplemented with 2 mM CaCl_2_ 2 mM MgSO_4_ (pH 7.4) and incubation at 37°C. At indicated time points cells were washed twice with PBS, fixed with 4% paraformaldehyde/sucrose solution and permeabilized for 5 min with 0.2% Triton X-100 in PBS. Coverslips were blocked for 1 h with 10% BSA in PBS and stained with EEA-1 antibody (1∶1000 in 1% BSA in PBS) for 1 h followed by staining with Alexa-Fluor 594 conjugated goat anti-mouse secondary antibody (1∶5000 in 1% BSA in PBS) for 45 min. Simultaneously, nuclei were counterstained with DAPI and coverslips were mounted onto glass slides and subjected to confocal laser scanning microscopy (Leica Inverted DM IRE 2).

### Neutralizing experiment

CHO-C6 cells were grown in 24-well chambers for 24 h under standard conditions. Prior to the experiment, 100 µl of 20 nM TcdA or TcdA^1–1874^, respectively, were diluted 1∶1 either with PBS or with antiserum α-TcdA_1875–2710_ and incubated for 20 min at RT in a rotating manner. Following pre-treatment, toxin/PBS- and toxin/antiserum-mixtures, respectively, were applied to the cultivated CHO-C6 cells at final concentration of 1 nM. Cell rounding of the cells was monitored by light microscopy as marker for toxin uptake and activity. After incubation for 5 h at 37°C, cytopathic effect was quantified as round cells per total cells [%]. Values are given as means ± SD, n = 5.

### Western blotting

Protein samples were separated by SDS-PAGE and transferred onto nitrocellulose membrane. After blocking with 5% (w/v) nonfat dry milk in TBST (50 mM Tris HCl pH 7.2, 150 mM NaCl, 0.05% (v/v) Tween-20) the membrane was incubated overnight with the primary antibody at 4°C. Following washing with TBST it was incubated for 1 h at room temperature with horseradish peroxidase conjugated secondary antibody (Rockland, USA). Detection was performed by means of chemiluminescence.

### TER measurement

CaCo-2 cells were seeded onto 12 well filter inserts (Transwell, pore size 0.4 µM, (Becton Dickinson, Germany) to measure the transepithelial electrical resistance (TER). Monolayers were cultivated up to an initial resistance of >150 Ω*cm^2^. The TER was determined by epithelial Voltohmmeter (EVOM, World Precision Instruments, Germany) equipped with Endom 24 chamber. To investigate toxin-induced deregulation of the intestinal barrier function, cells were apically or basolaterally treated with 1 nM of TcdA and TcdA^1–1874^ or 100 pM of TcdB and TcdB^1–1852^, respectively, and TER was measured at indicated time points. Values are given as means±standard deviations (N = 3).

### Ethics Statement

This study was conducted in accordance with German law for animal protection and with the European Communities Council Directive 86/609/EEC for the protection of animals used for experimental purposes. All protocols were approved and experiments were permitted by the local government (permission No. 33-42502-03A351 by the Lower Saxony State Office for Consumer Protection and Food Safety, LAVES, Oldenburg, Germany) and approved by the Local Institutional Animal Care and Research Advisory committee represented by the institutional animal welfare officer and head of the Institute for animal Science, Hannover Medical School, Prof. Dr. Hans-J. Hedrich.

## Results

### The CROP domain of TcdA is not solely responsible for toxin uptake

**Figure 1:**
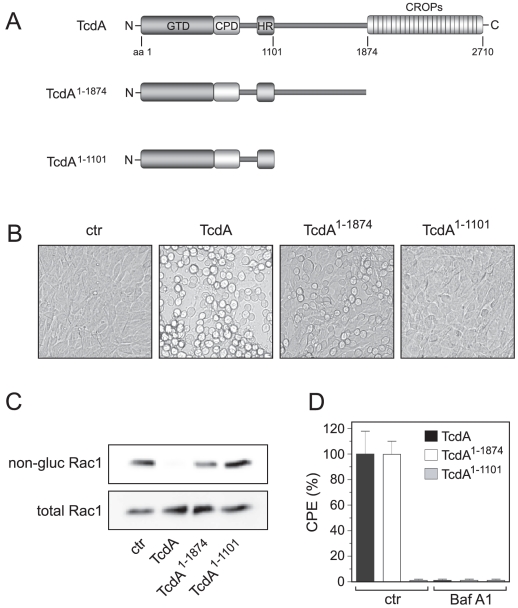
TcdA^1–1874^ lacking the C-terminal repeats still possesses cytotoxic potency. A) Multidomain structure of *C. difficile* TcdA and TcdA mutants TcdA^1–1874^ and TcdA^1–1101^. Full length TcdA consists of the N-terminal glucosyltransferase domain (GTD), the cysteinprotease domain (CPD), the hydrophobic region (HR) acting as transmembrane domain and the C-terminal combined repetitive oligopeptides (CROPs). The CROPs were deleted in TcdA^1–1874^. Mutant TcdA^1–1101^ exhibits the whole N-terminal domain including the hydrophobic region. B) Cell rounding assay of 3T3 fibroblasts after 90 min of toxin treatment with 1 nM of TcdA, TcdA^1–1874^ or TcdA^1–1101^, respectively. C) Western blot analysis of toxin-treated cells using antibody either recognizing only non-glucosylated (upper panel) or total Rac1 (lower panel). D) Pre-treatment of 3T3 fibroblasts with 100 nM of Bafilomycin A1, an inhibitor of endosomal acidification, prevents cell rounding of TcdA and TcdA^1–1874^ revealing specific cellular uptake of CROP-truncated TcdA. Cells were treated with equipotent concentrations of TcdA (1 nM) and TcdA^1–1874^ (10 nM) for 2 h.

Based on the literature we dispute the necessity of the C-terminal repeats for toxin functionality. Therefore we generated mutant TcdA lacking the supposed receptor binding domain (TcdA^1–1874^) and investigated the cytopathic impact towards host cells in cell rounding and Rac1-glucosylation assay (Fig. 1 B,C). Although lacking the receptor binding domain, TcdA^1–1874^ (1 nM) possesses cytopathic potency when applied to 3T3 fibroblasts. Instead, mutant TcdA^1–1101^, consisting of the N-terminal domain and the hydrophobic region, did not induce cell rounding or Rac1-glucosylation excluding non-specific uptake of TcdA^1–1874^ (Fig. 1B). Even at 300-fold higher concentration (300 nM) TcdA^1–1101^ did not show any signs of cytotoxicity after 48 h (data not shown). In addition, Bafilomycin A1, an inhibitor of endosomal acidification, prevented TcdA^1–1874^-induced cytopathic effects reflecting that this CROP-deletion mutant is also internalized by receptor-mediated endocytosis (Fig. 1D).

**Figure 2:**
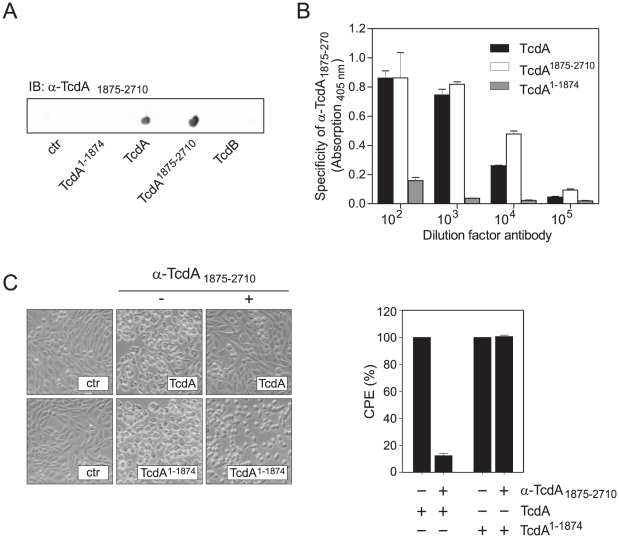
Neutralization assay emphasizes the role of TcdA CROPs in toxin functionality. A) Dot blot showing specificity of generated polyclonal antiserum α-TcdA_1875–2710_. The antiserum raised against the CROPs of TcdA only recognizes full length TcdA as well as the isolated CROP domain TcdA^1875–2710^. No cross-reactivity was detected towards CROP-truncated TcdA^1–1874^ or native TcdB, respectively. *B. megaterium* lysate was used as negative control. B) Recognition of full length toxin or toxin fragments by α-TcdA_1875–2710_ was checked by ELISA. A 96-well plate was coated with full length TcdA, TcdA^1–1874^ and TcdA^1875–2710^, respectively. The bar diagram shows absorption at 405 nm after concentration-dependent binding of α-TcdA_1875–2710_ to full length TcdA and the C-terminal repeats. Binding to TcdA^1–1874^ was only observed to a small extent and after applying high amounts of antiserum (dilution factor 1∶100). Values are given as means ± standard deviation, n = 5. C) Neutralization of TcdA and TcdA^1–1874^ with α-TcdA_1875–2710_ antiserum was investigated in CHO-C6 cell rounding assay (left panel). Cytopathic effect (CPE) was quantified as round cells per total cells in %. Values are given as means ± SD, n = 5 (right panel).

**Figure 3:**
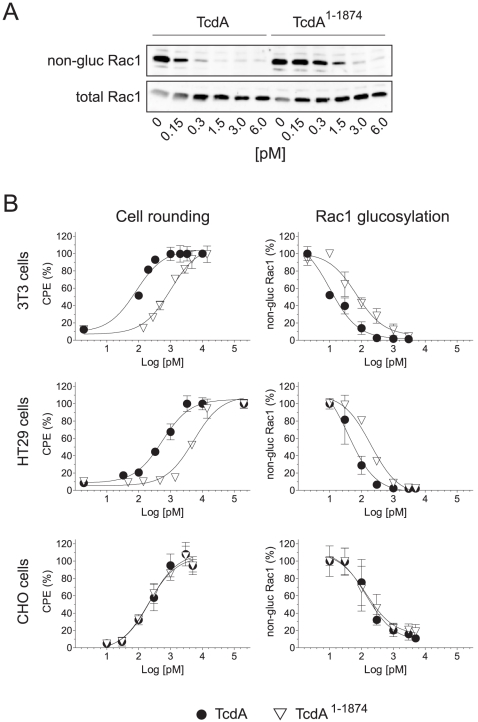
Cytotoxic potency of TcdA and TcdA^1–1874^ towards host cells. A) HT29 cells were treated with full length TcdA or TcdA^1–1874^ in a concentration-dependent manner until onset of cell rounding. Western blot analysis was performed to monitor level of glucosylated Rac1 using antibodies recognizing either non-glucosylated Rac1 (upper panel) or total Rac1 (lower panel), respectively. B) Dose-dependent analysis of cytopathic effect (CPE) and Rac1-glucosylation induced by TcdA (•) and TcdA^1–1874^ (▿) on 3T3, HT29 and CHO-C6 cells. Cytopathic effect was quantified as round cells per total cells in %. Results of Rac1-glucosylation are based on immunoblot analyses exemplarily shown in A). Values are given as means ± SD, n = 3.

To further evaluate the role of the C-terminal repeats, neutralizing experiments with polyclonal antiserum raised against the amino acids 1875–2710 were performed. Dot blot analysis and ELISA confirmed specificity of α-TcdA_1875–2710_ (Fig. 2A,B). As supposed, α-TcdA_1875–2710_ prevented the cytopathic effect of full length TcdA over the observed period of 5 h (Fig. 2C) implying a crucial role for the repetitive sequences with regard to toxin functionality. Interestingly, the strong cytopathic effect induced by TcdA^1–1874^ was not affected by this antiserum strengthening the hypothesis that the CROPs do not constitute the sole receptor binding domain. To further characterize the CROP-independent impact full length and truncated TcdA were compared in cell rounding assays to measure their cytopathic potency and with respect to intracellular glucosylation of Rac1. Glucosylation of Rac1 was indirectly detected by Western blot with specific antibody only recognizing non-glucosylated Rac1 [18]. Fig. 3A exemplarily shows concentration-dependent decrease of non-glucosylated Rac1 levels in comparison to total Rac1 of HT29 cells. Dose- dependent rounding of cells (cytopathic effect, CPE) showed half-maximum effect (EC_50_) at a concentration of 0.55 nM for full length TcdA and 5.3 nM for the mutant TcdA^1–1874^ (Fig. 3B, middle panel). Rac1-glucosylation was 5-fold reduced for TcdA^1–1874^ compared to full length TcdA. The discrepancy between CPE and Rac1-glucosylation is due to the need of almost quantitative Rac1-glucosylation to achieve complete cell rounding. Similar results were found for 3T3 fibroblasts, although these cells were 5-fold more sensitive than HT29 cells. Compared to full length TcdA, the cytopathic potency of TcdA^1–1874^ towards 3T3 fibroblasts was about 10-fold reduced with respect to cell rounding assay (EC_50_: 83 pM *vs.* 989 pM) and about 5-fold reduced with respect to Rac1 glucosylation (EC_50_: 8 pM *vs.* 41 pM). Interestingly, CHO-C6 cells show almost identical susceptibility towards full length and truncated TcdA regarding cytopathic effect (EC_50_: 223 pM *vs.* 203 pM) and Rac1-glucosylation status (EC_50_: 130 pM *vs*. 121 pM). All data are summarized in table 1.

**Table 1:** EC_50_ [pM] of TcdA and TcdA^1–1874^ regarding cytopathic effect and Rac1-glucosylation.

	Cytopathic effect		Rac1 glucosylation	
EC_50_ [pM]	TcdA	TcdA^1–1874^	TcdA	TcdA^1–1874^
**3T3**	83	989	10	68
**HT29**	549	5309	41	198
**CHO**	223	203	130	121

**Figure 4:**
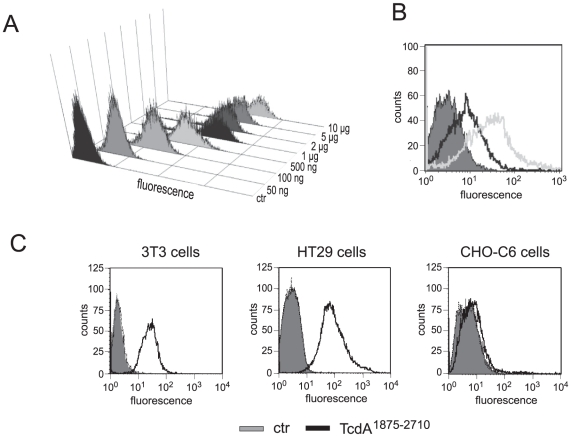
Binding of TcdA CROPs to host cells. A) Suspended HT29 cells were incubated at 4°C with increasing amounts of EGFP-labelled TcdA^1875–2710^ to determine optimal protein-to-cell ratio for binding studies. FACS analysis revealed saturation of binding at 1 µg protein per 500,000 cells. B) HT29 cells were pre-treated with 5 µg/ml non-labelled TcdA^1875–2710^ followed by incubation with 1 µg/ml EGFP-labelled TcdA^1875–2710^ to examine binding specificity of the fusion protein by FACS analysis. Right shift of the light grey curve to higher fluorescence reflects binding of EGFP-TcdA^1875–2710^ to HT29 cells. Competition with non-labelled CROP domain reduced binding (black curve) revealing same specificity of the fusion protein. EGFP alone was used as negative control (dark grey). C) FACS analysis with EGFP (grey curve) or EGFP-labelled TcdA CROP domain (black curve) was performed to study CROP binding to different host cells. As shown by right shift of the black curve TcdA CROPs strongly bind to 3T3 and HT29 cells whereas faint binding was monitored to CHO-C6 cells.

We additionally performed FACS-analysis with EGFP-fused CROPs to investigate binding of the repetitive sequences to different host cells. In order to determine the amount of protein necessary to saturate binding sites, HT29 cells were incubated with increasing amounts of EGFP-TcdA^1875–2710^. FACS analysis revealed that saturation of binding was achieved by application of 1–2 µg protein to 500,000 cells (Fig. 4A). To examine binding specificity of the EGFP-fused CROP domain, competition assay was performed. Fig. 4B nicely shows that pre-incubation of HT29 cells with non-labeled CROPs reduced the amount of bound EGFP- TcdA^1875–2710^ dramatically indicating that EGFP-labeling does not alter binding properties of the CROPs. We therefore used this fusion protein for binding studies to host cells and found that the C-terminal repeats of TcdA strongly bound to 3T3 and HT29 cells, as shown by curve shift in Fig. 4C (left and middle panels, black curve). Interestingly, no binding of the CROPs to CHO-C6 cells was observed. This is in accordance with cell rounding and Rac1-glucosylation assays where CHO-C6 cells were as sensitive to full length as to truncated TcdA^1–1874^ (compare Fig. 3, lowest panel). Similar to the effects observed for TcdA, CROP-deleted TcdB (TcdB^1–1852^) is still cytopathic towards all used cell lines (data not shown). However, in any case TcdB was more potent than TcdB^1–1852^. These data nicely indicate that repeats in the C-terminus of TcdA modulate the potency of TcdA and TcdB towards a variety of cells. There are, however, exceptions where the CROPs of TcdA do not develop this effect, as shown by treatment of CHO-C6 cells. Towards these cells, full length TcdA and truncated TcdA possess comparable potency. Thus, CROP-binding to the cell surface basically correlates with toxin potency. These findings refute the accepted opinion of a solely CROP- mediated toxin uptake. Toxin internalization into host cells might additionally occur through an alternative receptor structure and/or corresponding endocytotic pathway.

### The CROP domain triggers high rated endocytosis

**Figure 5:**
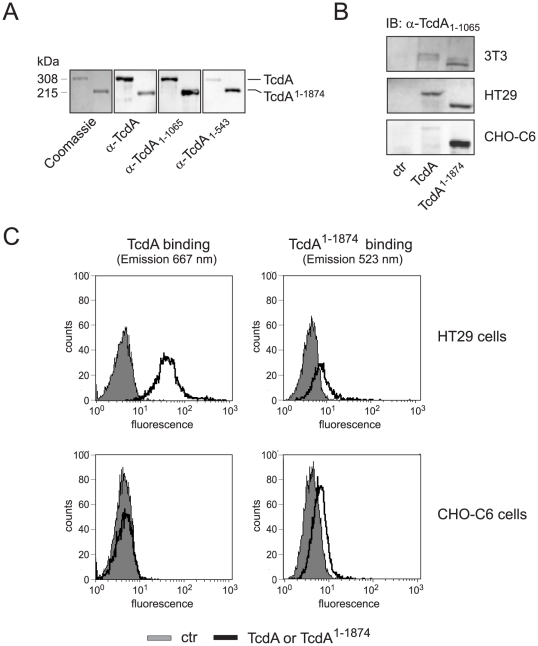
Binding of TcdA and TcdA^1–1874^ to host cells. A) The polyclonal antisera α-TcdA_1–2710_, α-TcdA_1–1065_ and α-TcdA_1–543_ were tested by Western blot analyses to identify a specific antibody detecting full length and truncated TcdA with same sensitivity. Antiserum α-TcdA_1–1065_ was selected for further studies showing strong and comparable recognition of TcdA and TcdA^1–1874^. B) Binding of TcdA and TcdA^1–1874^ to intact 3T3, HT29 and CHO-C6 cells was performed for 30 min at 4°C and analyzed by Western blot with α-TcdA_1–1065_. C) Binding of fluorescent labeled TcdA-PE/Cy5 and TcdA^1–1874^-Atto488 to HT29 and CHO-C6 cells was investigated by FACS analysis. Right shift of the black curve illustrates toxin binding which was detected through fluorescence emission at 667 nm for TcdA and at 523 nm for TcdA^1–1874^, respectively. Due to different ratio of fluorophor and toxin, fluorescence intensity of TcdA-PE/Cy5 cannot directly be compared with TcdA^1–1874^-Atto488.

Since the approach of binding studies of the isolated, epitope-labelled domains to suspended cells is very artificial we further characterized and compared binding of the full length and truncated TcdA to cell surfaces of intact cells. In order to identify a specific antibody detecting full length and truncated TcdA with same sensitivity, different polyclonal antisera were tested by Western blot analyses. Fig. 5A displays strong and comparable recognition of TcdA and TcdA^1–1874^ by antiserum α-TcdA_1–1065_, for which reason this antibody was used for further studies.

**Figure 6:**
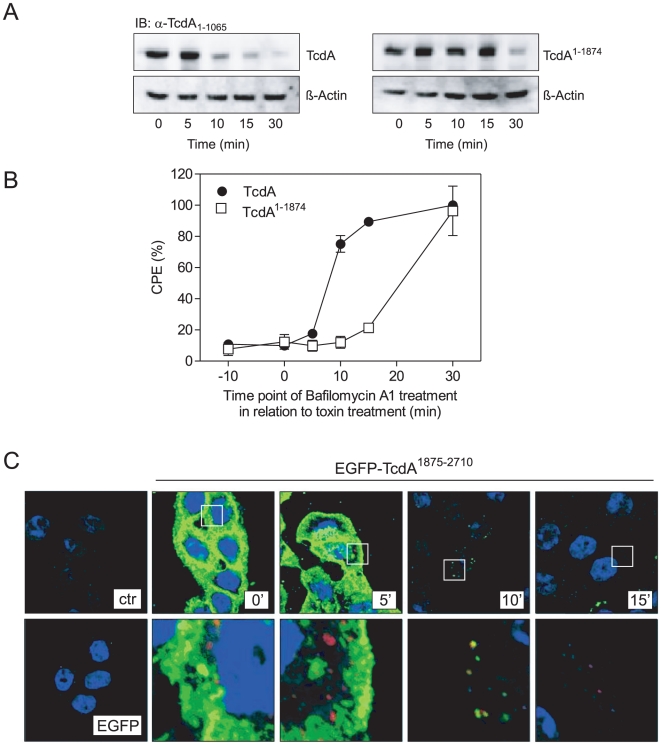
Cellular uptake of TcdA, TcdA^1–1874^ and TcdA^1875–2710^. A) Efficiencies of TcdA and TcdA^1–1874^ uptake into HT29 cells were indirectly determined by monitoring lysosomal toxin degradation following endosomal acidification. After binding at 4°C, endocytosis of toxin was allowed by temperature shift to 37°C. At the indicated time-points cells were lyzed and lysates were subjected to Western blot analysis to detect non-degraded toxins. β-Actin served as control protein excluding non-specific protein degradation. B) TcdA or TcdA^1–1874^ was applied to 3T3 fibroblasts (time point 0) and Bafilomycin A1 was added at indicated times before or after toxin application. Cell rounding (CPE) was quantified 3 h after toxin treatment as rounded cells per total cells in %. Values are given as means ± SD (N = 3). C) Endocytosis of the isolated TcdA CROP domain (TcdA^1875–2710^) was proven by immunofluorescence microscopy. Binding of EGFP or EGFP- labeled TcdA^1875–2710^ to HT29 cells were performed on ice followed by a temperature-shift to 37°C allowing endocytotic processes. At indicated time points, cells were washed and fixed and immunofluorescence microscopy was performed to display nuclei (DAPI, blue), TcdA^1875–2710^ (EGFP, green) and early endosomes (EEA1, red). Untreated (ctr) and EGFP-bound cells (EGFP) were used as controls. The lower panels illustrate magnification of an area indicated by rectangles in the upper panels.

It is noteworthy that in immunoblot analyses full length and C-terminal deleted TcdA bind in a comparable manner to 3T3 or HT29 cells (Fig. 5B) though monitoring an almost 10-fold reduced CPE of TcdA^1–1874^ compared to full length TcdA (compare Fig. 3). In order to support this observation, binding was additionally analyzed by flow cytometry with fluorescent labeled toxins (Fig. 5C). Labeled toxins still possess cytopathic activity as checked by cell rounding and Rac1 glucosylation assays (data not shown). This approach also confirmed specific binding of full length as well as of CROP-truncated TcdA to HT29 cells. The difference in fluorescence intensities is a result of different fluorescent labeling of TcdA and TcdA^1–1874^ (TcdA-PE/Cy5 and TcdA^1–1874^-Atto488) as well as of different labeling ratio due to size of toxins. Thus, these data should be interpreted qualitatively. Both, TcdA as well as TcdA^1–1874^, bind to HT29. Interestingly, as confirmed by both approaches, CHO cells showed enhanced binding of truncated TcdA and no binding of the full length protein (Fig. 5B, bottom panel and Fig. 5C, lower panel) though being identical susceptible towards both toxins (compare Fig. 3). These findings imply that TcdA^1–1874^ binds to abundant receptor structures with low uptake rate whereas CROP- specific binding structures, indeed, are rare but ensure potent uptake. To substantiate this hypothesis, uptake efficiencies of TcdA and TcdA^1–1874^ into host cells were compared. Therefore, time-dependent lysosomal toxin degradation following endosomal acidification was monitored as indirect marker for endocytosis rate. Following binding of TcdA and TcdA^1–1874^ at 4°C to HT29 cells, endocytotic toxin-uptake was induced by temperature-shift to 37°C. At the indicated time-points cells were lyzed and lysates were subjected to Western blot analysis to detect non-degraded toxins. In fact, degradation of TcdA^1–1874^ occurred with a marked delay compared to TcdA revealing a faster endocytotic process of the full length toxin (Fig. 6A). This notion was corroborated by another approach. Bafilomycin A1 was applied to HT29 cells at different time points after treatment with TcdA or TcdA^1–1874^ to inhibit endosomal acidification. As nicely shown in Fig. 6B, endocytosis of full length TcdA was prevented by Bafilomycin A1 only when applied within 5 min after toxin treatment. Inhibition of endocytosis 10 min after toxin treatment did not prevent TcdA uptake, as monitored by 80% of cell rounding (Fig. 6B, •). Instead, endocytosis of truncated TcdA^1–1874^ could still be inhibited (at least for 3 h of incubation) by Bafilomycin A1 when applied 15 min after toxin treatment (Fig. 6B, □). These data are in good agreement with immunoblot analyses shown in Fig. 6A.

Interestingly, confocal microscopy revealed comparable time-dependent uptake of EGFP-labeled CROP domain (EGFP-TcdA^1875–2710^) compared to full length TcdA. Five minutes following temperature shift, EGFP-TcdA^1875–2710^ strongly bond to the surface of HT29 cells (Fig. 6B, green). After 10 min of incubation, CROPs were almost completely endocytosed as monitored by disappearance of green fluorescence at the cell surface. In addition, EEA1 were stained to visualize early endosomes. Simultaneous emergence of yellow spots inside the cells reflects EGFP-labeled CROPs co-localized with EEA1 in early endosomes after 10 min. Continuous lysosomal degradation of internalized TcdA fragments was monitored by a reduction of co-localized signals after 15 min accompanied by appearance of red-stained recycled early endosomes. This finding is in accordance with uptake rates determined by Western blot analysis for full length TcdA (Fig. 6A, left panel). We concluded that uptake of TcdA might be predominantly mediated by the C-terminal repeats ensuring potent toxin internalization. Cellular uptake of CROP-truncated TcdA^1–1874^ occurred with a marked delay supporting the notion of at least one alternative route that can be used by the toxin.

### Cytopathic potencies of full length and CROP-deleted toxins A and B towards polarized cells depend on the site of application

**Figure 7:**
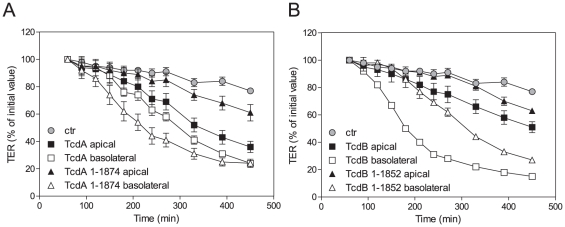
Effect of full length and CROP-deleted C. difficile toxins on intestinal barrier function following apical or basolateral uptake. A) 1 nM of TcdA (□,▪) and TcdA^1–1874^ (Δ,▴) and B) 100 µM of TcdB (□,▪) and TcdB^1–1852^ (Δ,▴) were applied to the apical (filled symbols) or basal (open symbols) compartment of CaCo-2 cells grown on Transwell filter inserts. Transepithelial electrical resistance (TER) was monitored over time as marker for toxin-uptake and activity. Untreated cells were used as control. Values are given as % of initial value after equilibration as means ± SD, n = 3.

To further investigate an alternative internalization mechanism for CROP- truncated *C. difficile* toxins, toxin uptake into polarized CaCo-2 cells was analyzed. CaCo-2 cells serve as model for different receptor structures and/or protein composition of the apical and basolateral membrane. Toxin-induced disturbance of the intestinal barrier function, monitored by reduction of transepithelial electrical resistance (TER), was measured as marker for toxin internalization. In coherence with previous data [19], TcdA possesses almost identical cytotoxicity when applied apically or basolaterally (Fig. 7A □,▪) whereas cytotoxic potency of TcdB strongly depends on the site of application (Fig. 7B □,▪). Interestingly, basolaterally applied TcdA^1–1874^ exhibits considerably higher cytotoxic potency than apically applied or even full length TcdA. This observation was confirmed by intracellular Rac1-glucosylation (data not shown). In contrast to TcdA, TcdB was more potent than its truncated form TcdB^1–1852^, independent on the site of application. Thus, the CaCo-2 model is a cell line where different endocytotic routes are present and can be studied separately. These findings strongly speak in favor for different uptake routes than for cell specific differences in endocytosis of toxins.

### TcdA and TcdA^1–1874^ did not compete for cellular receptors

**Figure 8:**
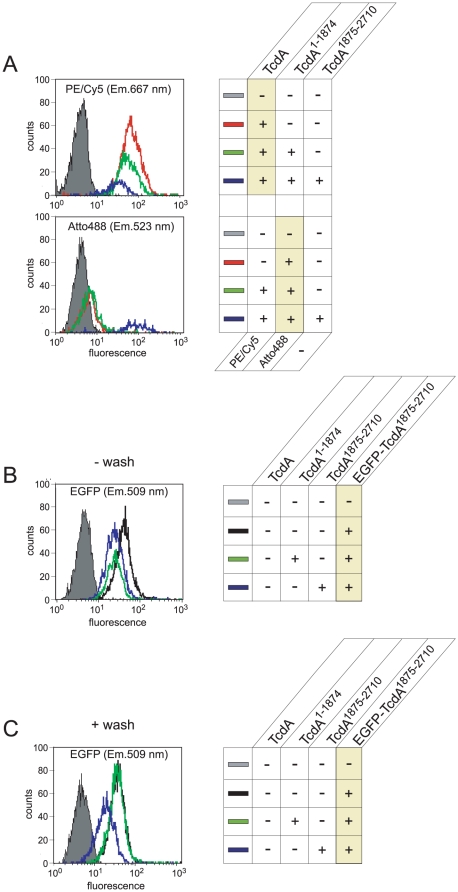
Competition of TcdA, TcdA^1–1874^ and TcdA^1875–2710^ for cellular binding structures. A) Binding competition of PE/Cy5-labeled TcdA, Atto488-labeled TcdA^1–1874^ and EGFP-fused TcdA^1875–2710^, respectively, for cellular receptor structures was analyzed by flow cytometry. Therefore, 4 nM of TcdA-PE/Cy5 (red curve, upper panel) or TcdA^1–1874^-Atto488 (red curve, lower panel) were applied either separately, in combination (green curves) or simultaneously after pre-incubation with 150 nM non-labeled TcdA^1875–2710^ (blue curves) to HT29 cells. Intensity of fluorescence emission (x-axis) at 667 nm and 523 nm illustrates binding of TcdA and TcdA^1–1874^ to HT29 cells, respectively. B) HT29 cells were treated with 150 nM of TcdA^1875–2710^ (black curve) or pre-incubated with either 150 nM of TcdA^1–1874^ (green curve) or TcdA^1875–2710^ (blue curve) followed by immediate incubation with 8 nM of EGFP-fused TcdA^1875–2710^ in the presence of either toxin. FACS analysis of EGFP-induced fluorescence emission at 509 nm showed reduced binding of CROP-truncated TcdA^1–1874^ (compare black and green curve). C) The same experiment as shown in B) except that excessive toxin was washed off before addition of EGFP-fused TcdA^1875–2710^. These results indicate that excessive TcdA^1–1874^ sequesters TcdA^1875–2710^ in solution being falsely interpreted as competition. The yellow highlighted lanes in legends mark the fluorescence labeled toxins that were detected at wavelength shown in respective graphs.

To further elucidate the cellular binding structures of full length and truncated TcdA, competition experiments were performed and evaluated by flow cytometry. Therefore, fluorescent labeled TcdA and TcdA^1–1874^ were applied to HT29 cells either separately, in combination or simultaneously after saturation of the cell surface structures with TcdA^1875–2710^ (Fig. 8A). Fluorescence emission at 667 nm (Fig. 8A, upper panel) and 523 nm (Fig. 8A, lower panel) revealed binding of TcdA and TcdA^1–1874^, respectively (red curves). Interestingly, presence of TcdA apparently did not affect binding of TcdA^1–1874^ to HT29 cells (lower panel, green curve). This finding implies that truncated TcdA utilizes other receptor structures for cellular uptake than the full length toxin. However, a slightly reduced binding capacity of TcdA was observed in the presence of its truncated form (upper panel, compare green and red curves). As expected, the isolated TcdA CROPs (TcdA^1875–2710^) clearly compete with the full length toxin for binding structures at the cell surface of HT29 cells (upper panel, compare blue and red curves). Surprisingly, pre-incubation with TcdA^1875–2710^ dramatically increased fluorescence intensity emitted from Atto488- labeled TcdA^1–1874^ (lower panel, blue curve) implying enhanced binding of TcdA^1–1874^ to HT29 cells. It is conceivable that this phenomenon is due to binding of truncated toxin to the isolated CROP domain immobilized at the cell surface. This hypothesis is supported by an experiment illustrated in Fig. 8B. HT29 cells were saturated with either TcdA^1875–2710^ or TcdA^1–1874^ followed by immediate addition of EGFP-fused TcdA^1875–2710^. Following incubation, cells were washed and analyzed by flow cytometry. As expected, the isolated TcdA CROPs compete with the EGFP-labeled CROP domain (Fig. 8B, compare blue and black curves). Surprisingly, this seemed to be true for truncated TcdA^1–1874^ as well (compare green and black curves). In accordance with the conclusion drawn from Fig. 8A, we hypothesized that the excess of TcdA^1–1874^ sequestered the CROPs in solution resulting in a signal reduction of cell-bound TcdA^1875–2710^ after washing. This assumption was confirmed by a second approach in which the excessive putative competitor TcdA^1–1874^ was washed off the cells before subsequently the TcdA CROPs were allowed to bind the respective receptor structures. In fact, the apparent competition between TcdA^1–1874^ and TcdA^1875–2710^ disappeared under this condition, emphasizing that full length and CROP-truncated TcdA bind different surface structures.

## Discussion

The current study investigates the role of the C-terminal repeats of *C. difficile* TcdA regarding toxin functionality. The report by Amimoto [13] showed that the novel identified TpeL from *C. perfringens*, which is homologous to large clostridial glucosylating toxins, possesses cytotoxic activity. This toxin, however, lacks the C-terminal repeats that are typical for clostridial glucosyltransferases and serve as receptor binding domain. Based on this finding we dispute the necessity of the CROP domain concerning functional properties of the *C. difficile* toxins A and B. By recombinant expression and purification of full length TcdA and truncated TcdA^1–1874^ we were able to compare the cytopathic impact of both TcdA forms. Indeed, CROP-truncated TcdA^1–1874^ induced time- and concentration dependent rounding of host cells. Since the N-terminal- and transmembrane domain-covering mutant TcdA^1–1101^ was completely inactive in cell rounding and Rac1 glucosylation assays and inhibition of endosomal acidification prevented TcdA^1–1874^-induced effects, we excluded non-specific cellular uptake. This was in line with previously reported lack of cytotoxic potency of TcdA^1–1065^ [20].

Recently, Demarest and co-workers described potent toxin neutralization by a combination of monoclonal antibodies directed against multiple sites of TcdA localized within the proposed receptor binding domain [21]. This is in absolute accordance with our findings and the question arises why antibody solely directed against the C-terminal repeats is able to neutralize the effect of TcdA. This phenomenon might be explained by simultaneous steric inhibition of the vicinal intermediate domain or by hindering conformational changes of the domains as a prerequisite for binding to the functional receptor.

In this study we clearly show that TcdA can display its cytopathic action in the absence of the C- terminal repeats. This finding is new and puts light on the intermediate and transmembrane domain as target for further receptor interaction. It can be assumed that the same is true for TcdB. In coherence with our data, Barroso and co-workers found that the cytotoxic properties of *E. coli* lysates transfected with the corresponding truncated *tcdB* gene was about 10-fold reduced compared to lysates from full length TcdB transfected ones [22]. Despite the nature of the toxin itself, our data indicate that different cells are differently sensitive to TcdA because of the interaction with the C-terminal repeats. As can be deduced from EC_50_-values of Rac1-glucosylation (see Tab. 1), which is a direct marker of intracellular toxin action, the potency of TcdA lacking the CROPs differs less than 3-fold with respect to different cell types and species. In contrast, full length TcdA varies 3–10-fold in its potency, pointing out that sensitivity of cells towards TcdA is primarily defined by the interaction with the CROPs. This notion is supported by data from flow cytometry revealing that CROP binding to host cells correlates with increased potency of TcdA compared to truncated TcdA^1–1874^.

Binding of TcdA^1–1874^ was analyzed by Western blots. Although TcdA^1–1874^ was 10-fold less cytopathic compared to TcdA, binding of both toxins to HT29 cells was comparable. This discrepancy was even more obvious in CHO cells which exhibit enhanced binding of truncated TcdA with identical susceptibility towards both toxins. This raises the question about correlation of cell surface binding and endocytotic uptake. Comparative analyses of endocytosis efficiencies revealed that the described phenomenon is most likely due to a faster internalization process of full length TcdA compared to truncated TcdA^1–1874^. Confocal microscopy revealed that the TcdA CROPs alone are sufficient in triggering efficient endocytosis which occurred with rates almost identical to those observed by full length TcdA (Fig. 6). From these findings we concluded that uptake of full length TcdA is predominantly mediated by the C-terminal repeats. Most likely, TcdA^1–1874^ abundant receptor structures with low uptake rate whereas CROP- specific binding structures are less abundant but ensure potent internalization. The weak binding of full length TcdA to many cell surfaces compared to the truncated toxin might reveal that the CROPs mask alternative binding structures of the native toxin. This hypothesis is supported by a very recent publication of Pruitt and co-workers who elucidated variability in the structural organization of the functional toxin domains in pH- dependence [23]. New perceptions strongly indicate that the glucosyltransferase domain interacts with the C- terminally located CROPs and undergoes significant conformational changes following endosomal acidification leading to uncovering of the proposed alternative binding structures. It is conceivable that exposition and binding of these structures to the endosomal membrane is a prerequisite for translocation. Since cholesterol was shown to be essential for TcdA- and TcdB- mediated pore formation [24], it might be conceivable that a cholesterol binding region is sufficient for cell attachment allowing subsequent endocytosis. We previously reported that toxin fragments that encompass the CROPs plus a great part of the intermediate domain show stronger competition with full length TcdA than the mere CROPs [25]. That study nicely support that the CROPs are not solely responsible for binding and uptake of at least TcdA. The question, however, arises whether full length and truncated TcdA utilize different receptor structures and/or different routes for cellular uptake. We therefore investigated potential competition between TcdA, TcdA^1–1874^ and TcdA^1875–2710^ for cell binding by FACS analyses. Interestingly, neither full length TcdA nor the isolated CROPs compete with the truncated toxin for binding sites at HT29 cells indicating that TcdA^1–1874^ and TcdA^1875–2710^ bind to different surface structures (Fig. 8). Rather, pre-incubation with TcdA^1875–2710^ resulted in enhanced fluorescence intensity emitted from truncated TcdA which might be based either on binding of the N-terminal domain of TcdA^1–1874^ to the immobilized CROPs or to a potentially activated receptor. The first assumption refers to the study of Pruitt and co-workers who described an interaction of the glucosyltransferase domain of TcdA to its repetitive sequences at neutral pH [26]. Since CROP-truncated TcdA lacks its auto-ligand, the N-terminus might interact with CROPs immobilized at the cell surface leading to the observed dramatic increase of fluorescence intensity. The observation of TcdA^1–1874^ sequestering the CROPs in solution additionally supports this hypothesis (Fig. 8B,C). Another reason for increased fluorescence intensity of truncated TcdA following pre-incubation with TcdA^1875–2710^ could be the nature of the receptor: Binding of the CROPs to the cell surface might induce conformational changes and activates the specific receptor. This might be a prerequisite for binding of TcdA^1–1874^ through binding sites located in the intermediate part of the toxin. Hence, uptake of full length TcdA might occur in a two-step process explaining the potent endocytosis and increased toxin potency observed towards many cells compared to the truncated toxin. Even if the hypothesis has to be examined in more detail, we conclude that TcdA and TcdA^1–1874^ predominantly bind to different but not independent receptor structures.

We further investigated the hypothesis that internalization of TcdA and/or TcdB additionally occur via alternative routes. This hypothesis was evaluated and substantiated by comparative analyses of the toxin-induced reduction of transepithelial electrical resistance following apical or basolateral toxin uptake into CaCo-2 cells. Basolaterally applied TcdB possesses considerably higher potency in destroying epithelial integrity of monolayer than apically applied TcdB. This observation implies that expression of the TcdB-specific receptor is more or less restricted to the basolateral membrane, as also suggested by Stubbe and co-workers [19]. This discrepancy in sensitivity of apical and basolateral membrane surfaces was also observed towards CROP-deleted TcdB (TcdB^1–1852^), although TcdB^1–1852^ was less potent than full length TcdB. Interestingly, this was not the case regarding TcdA. While potency of full length TcdA is almost independent of the site of application, basolateral membranes show considerable increased endocytotic capacity towards CROP-truncated TcdA, resembling those effects observed for TcdB. This finding emphasizes the assumption of an additional alternative uptake process, at least for TcdA, which still might be based either on the recognition of different receptor structures or on the use of other associated endocytotic pathways. Thus, different routes for cellular uptake might enable the toxins to enter a broader repertoire of cell types leading to the observed multifarious pathogenesis of *C. difficile*.

The current study proved that the C-terminal repeats (CROPs) of TcdA and TcdB are not essential for the toxins biological function, albeit determining the potency of the toxin by their interactions with cell surface structures. Furthermore, we monitored huge variations in toxin potency towards different cell types as well as between the applied toxin forms. It should be noted that, contrary to other analyzed cell lines, CHO-C6 cells show identical susceptibility towards CROP-deleted and full length TcdA whereas the CROP-truncated mutant of TcdB possesses less potency towards these cells compared to the full length toxin. This is important, because CHO cells are of hamster origin, and the Syrian hamster model is widely used for *C. difficile* infection models [27]. Thus, if CHO cells are representative for hamster cells including enterocytes and colonocytes, studies on the relevance of toxins only have model character [28] and their extrapolation to human pathogenicity is limited. Different cells/species may notedly differ in their sensitivity to TcdA and TcdB. In addition, fragments or isoforms of toxins devoid of only the C-terminal repeats are also pathogenic. The latter finding might be of epidemiologic relevance with respect to the increasing prevalence of TcdA^−^/TcdB^+^ *C. difficile* strains. These strains are designated as TcdA-negative though, regarding serogroup F-strains, mere lacking 1800 base pairs within the repetitive regions [29], [30]. The resulting truncated TcdA lacking short parts of the CROP domain is cytopathic and most likely accounts for the pathogenesis observed in the variant *C. difficile* strains.

## References

[1] Voth DE, Ballard JD (2005). Clostridium difficile toxins: mechanism of action and role in disease.. Clin Microbiol Rev.

[2] Jank T, Aktories K (2008). Structure and mode of action of clostridial glucosylating toxins: the ABCD model.. Trends Microbiol.

[3] Von Eichel-Streiber C, Laufenberg-Feldmann R, Sartingen S, Schulze J, Sauerborn M (1992). Comparative sequence analysis of the Clostridium difficile toxins A and B.. Mol Gen Genet.

[4] Von Eichel-Streiber C, Sauerborn M (1990). Clostridium difficile toxin A carries a C-terminal structure homologous to the carbohydrate binding region of streptococcal glycosyltransferase.. Gene.

[5] Ho JG, Greco A, Rupnik M, Ng KK (2005). Crystal structure of receptor-binding C-terminal repeats from Clostridium difficile toxin A.. Proc Natl Acad Sci U S A.

[6] Dingle T, Wee S, Mulvey GL, Greco A, Kitova EN (2008). Functional properties of the carboxy-terminal host cell binding domains of the two toxins, TcdA and TcdB, expressed by Clostridium difficile.. Glycobiology.

[7] Tucker KD, Wilkins TD (1991). Toxin A of *Clostridium difficile* binds to the human carbohydrate antigens I, X, and Y.. Infect Immun.

[8] Demarest SJ, Salbato J, Elia M, Zhong J, Morrow T (2005). Structural characterization of the cell wall binding domains of Clostridium difficile toxins A and B; evidence that Ca2+ plays a role in toxin A cell surface association.. J Mol Biol.

[9] Pothoulakis C, Gilbert RJ, Cladaras C, Castagliuolo I, Semenza G (1996). Rabbit sucrase-isomaltase contains a functional intestinal receptor for *Clostridium difficile* toxin A.. J Clin Invest.

[10] Na X, Kim H, Moyer MP, Pothoulakis C, LaMont JT (2008). gp96 is a human colonocyte plasma membrane binding protein for Clostridium difficile toxin A.. Infect Immun.

[11] Just I, Gerhard R (2004). Large clostridial cytotoxins.. Rev Physiol Biochem Pharmacol.

[12] Pruitt RN, Chagot B, Cover M, Chazin WJ, Spiller B (2009). Structure-function analysis of inositol hexakisphosphate-induced autoprocessing in Clostridium difficile toxin A.. J Biol Chem.

[13] Amimoto K, Noro T, Oishi E, Shimizu M (2007). A novel toxin homologous to large clostridial cytotoxins found in culture supernatant of Clostridium perfringens type C.. Microbiology.

[14] Burger S, Tatge H, Hofmann F, Just I, Gerhard R (2003). Expression of recombinant Clostridium difficile toxin A using the Bacillus megaterium system.. Biochem Biophys Res Commun.

[15] Jainchill JL, Aaronson SA, Todaro GJ (1969). Murine sarcoma and leukemia viruses: assay using clonal lines of contact-inhibited mouse cells.. J Virol.

[16] Hidalgo IJ, Raub TJ, Borchardt RT (1989). Characterization of the human colon carcinoma cell line (Caco-2) as a model system for intestinal epithelial permeability.. Gastroenterology.

[17] Huet C, Sahuquillo-Merino C, Coudrier E, Louvard D (1987). Absorptive and mucus-secreting subclones isolated from a multipotent intestinal cell line (HT-29) provide new models for cell polarity and terminal differentiation.. J Cell Biol.

[18] Genth H, Huelsenbeck J, Hartmann B, Hofmann F, Just I (2006). Cellular stability of Rho-GTPases glucosylated by Clostridium difficile toxin B.. FEBS Lett.

[19] Stubbe H, Berdoz J, Kraehenbuhl J-P, Corthésy B (2000). Polymeric IgA is superior to monomeric IgA and IgG carrying the same variable domain in preventing *Clostridium difficile* toxin A damaging of T84 monolayers.. J Immunol.

[20] Teichert M, Tatge H, Schoentaube J, Just I, Gerhard R (2006). Application of Mutated Clostridium difficile Toxin A for Determination of Glucosyltransferase-Dependent Effects.. Infect Immun.

[21] Demarest SJ, Hariharan M, Elia M, Salbato J, Jin P (2010). Neutralization of Clostridium difficile toxin A using antibody combinations.. MAbs.

[22] Barroso LA, Moncrief JS, Lyerly DM, Wilkins TD (1994). Mutagenesis of the *Clostridium difficile* toxin B gene and effect on cytotoxic activity.. Microb Pathog.

[23] Pruitt RN, Chambers MG, Ng KK, Ohi MD, Lacy DB (2010). Structural organization of the functional domains of *Clostridium difficile* toxins A and B..

[24] Giesemann T, Jank T, Gerhard R, Maier E, Just I (2006). Cholesterol-dependent pore formation of clostridium difficile toxin A.. J Biol Chem.

[25] Frisch C, Gerhard R, Aktories K, Hofmann F, Just I (2003). The complete receptor-binding domain of Clostridium difficile toxin A is required for endocytosis.. Biochem Biophys Res Commun.

[26] Pruitt RN, Chambers MG, Ng KK, Ohi MD, Lacy DB (2010). Structural organization of the functional domains of Clostridium difficile toxins A and B.. Proc Natl Acad Sci U S A.

[27] Bartlett JG, Chang TW, Gurwith M, Gorbach SL, Onderdonk AB (1978). Antibiotic-associated pseudomembranous colitis due to toxin-producing clostridia.. N Engl J Med.

[28] Lyras D, O'Connor JR, Howarth PM, Sambol SP, Carter GP (2009). Toxin B is essential for virulence of Clostridium difficile.. Nature.

[29] Kato H, Kato N, Katow S, Maegawa T, Nakamura S (1999). Deletions in the repeating sequences of the toxin A gene of toxin A-negative, toxin B-positive Clostridium difficile strains.. FEMS Microbiol Lett.

[30] van den Berg RJ, Class ECJ, Oyib DH, Klaassen CHW, Dijkshoorn L (2004). Characterization of toxin A-negative, toxin B-positive Clostridium difficile isolates from outbreaks in different countries by amplified fragment length polymorphism and PCR ribotyping.. J Clin Microbiol.

